# Drink and Accidents

**Published:** 1895-04-06

**Authors:** 


### The Hospital

An Institutional Journal of

The Medical Sciences and Hospital Administration, with

AN EXTRA NURSING SUPPLEMENT.

Vol. XVIII.—No. 445.] [April 6, 1895.

### Drink and Accidents.

The question of the manifold physical ills which are directly caused by the use of stimulants is always a "burning" one with that section of the public which tastes no alcohol. The interest taken in it is by no means limited to total abstainers. On the contrary, very important public issues are involved, and thoughtful persons will be glad of any undoubted facts which may throw a clear light on the subject.

The Honourable Sydney Holland, chairman of the committee of the Poplar Hospital for Accidents, put forward statements in a communication to the *Times* the other day which have been read by most people with surprise, and by many with absolute incredulity. We had been accustomed to think, perhaps largely in consequence of the unchastened rhetoric of the total abstinence platform, that a very large proportion—at least half—of all the accidents which daily and hourly occur in the streets of London are due either directly or indirectly to the drinking habits of our fellow countrymen. We have been all wrong, Mr. Sydney Holland tells us, as wrong as wrong could be. In his position of chairman of the chief of the "accident hospitals" of London he has had special opportunities for getting at the exact truth. He has utilised those opportunities to good purpose. During a period of six months, in which the large total of 8,276 accident cases have been received at the Poplar Hospital, careful records have been kept of the exact number which have been definitely due to drink. Instead of one half, Mr. Sydney Holland has found rather less than one in twenty-seven.

A result so surprising as this, however satisfactory it may be in itself, at least invites to further investigation. The Poplar Hospital is, after all, but one among many. It was natural to ask what might be the experience of other hospitals placed in different parts of London and treating somewhat different classes of patients from those treated at Poplar. Accordingly, the writer called upon the house surgeons at St. Bartholomew's, Charing Cross, Westminster, St. Thomas's, and Guy's Hospitals, and discussed with those gentlemen the pros and cons of Mr. Sydney Holland's contribution to the subject.

As may already have been anticipated, Mr. Sydney Holland's breezy optimism is by no means confirmed by the experience of other hospitals. The house surgeons, one and all, expressed great surprise at the statements put forward, and, on the first blush, declared emphatically that their own experience pointed in a different direction. At St. Bartholomew's, Mr. Forbes Fraser held very firm convictions. In his judgment, not one in twenty-seven, but one in four would probably be the proportion of accidents caused by drink. At Charing Cross, Mr. Escombe was less surprised than Mr. Fraser had been; but he was of opinion that if night accidents—that is, accidents occurring between midnight and six a.m.—were considered by themselves, probably as many as half of the whole number were due to drink alone. Night accidents constitute but a small proportion of cases in point of number, but in point of seriousness they are relatively more important than those which occur during the day. As Mr. Escombe pointed out, it is desirable to classify accidents, and to get at the proportions of those which are insignificant, those which are trivial, those which are serious, those which are dangerous, and those which are fatal. The number of casualties of an insignificant or trivial character which are taken daily to the London hospitals is astonishing, and if all such trifling cases are counted as "accidents" for the purposes of the enumerator, then Mr. Escombe thinks that a smaller proportion are due to drink than even that given by Mr. Sydney Holland. He suggests one in fifty rather than one in twenty-seven. But a great many such cases he would not dignify with the name of "accidents" at all.

At St. Thomas's, Mr. F. C. Abbott could go back upon a six years' experience in residence at the hospital. His testimony was, therefore, specially valuable. Mr. Abbott, after carefully considering the question, is of opinion that as many as "fifteen per cent. of accidents are directly due to drink, and if we include those caused by the drunkenness of others and not of the patient, the proportion would be somewhat higher." The "large majority of accidents in the day-time and on ordinary evenings are not due to drink," but the "busy scene in a hospital casualty room on a Saturday night is entirely due to this one cause." With these experiences those of Mr. Martin, at Westminster

Hospital, and Mr. Davies, at Guy's, generally coincided.

There is no desire on our part to question the facts put forward by Mr. Sydney Holland in connection with the Poplar Hospital for Accidents, but it is clear that they do not give a correct picture of what is happening at the metropolitan hospitals generally. They correct, it is true, our too exaggerated estimate of the mere numbers of accidents caused entirely by drink, but they do not present to us that Hogarthian spectacle which may be witnessed almost any night between midnight and early morning in the casualty wards of any one of our larger London hospitals, and which on Saturday night reaches a maximum of blood and butchery the humours of which only the pencil of a Hogarth could adequately portray. Far be it from us to wish to exaggerate the facts in ever so slight a degree. On the contrary, nobody would have rejoiced more than ourselves if the large general hospitals of the metropolis could have confirmed Mr. Sydney Holland's picture of roseate hue. But the facts are not so, and we must take the facts exactly as they are, and make the best we can of them.

